# Advancing clinical-basic-clinical research: exploring novel immunotargets for ovarian cancer

**DOI:** 10.1038/s41392-024-01970-6

**Published:** 2024-09-30

**Authors:** Yuanzhuo Gu, Long Zhang, Weiguo Lv

**Affiliations:** 1 Department of Gynecological Oncology, Women’s Hospital, Zhejiang University School of Medicine, 310006 Hangzhou, China; 2 MOE Key Laboratory of Biosystems Homeostasis & Protection and Innovation Center for Cell Signaling Network, Life Sciences Institute, Zhejiang University, 310058 Hangzhou, China

**Keywords:** Cancer therapy, Translational research, Cancer microenvironment

**Fig. 1:**
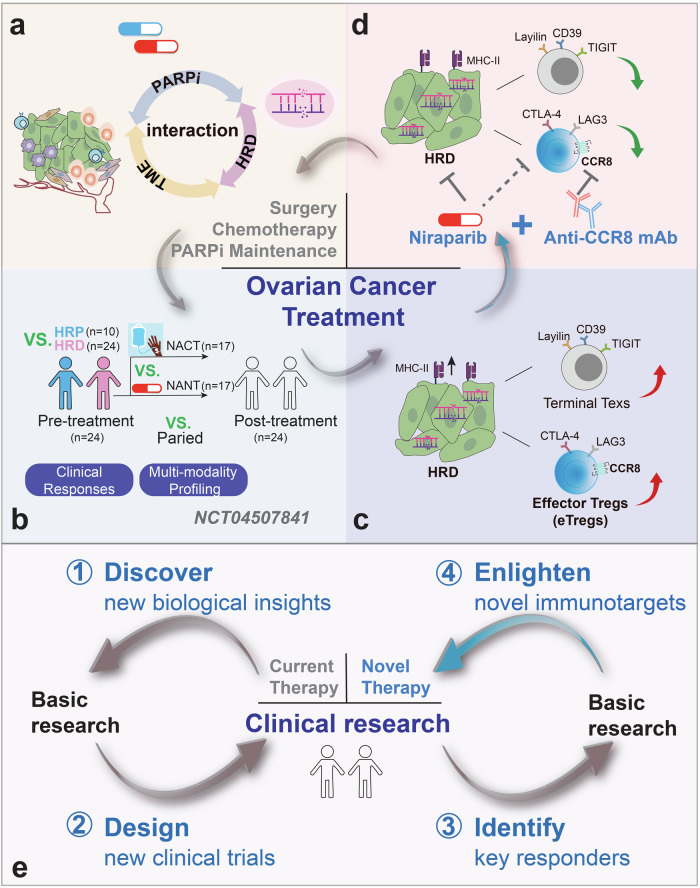
Schematic diagram of the clinical-basic-clinical cycle. The upper panel presents a specific case study exemplifying the integrative clinical-basic-clinical approach, which recently published in a *Cell* paper. **a** The current therapy for HRD ovarian cancer mainly includes surgery, platinum-based neoadjuvant chemotherapy and PARPi maintenance. On this basis, the authors hope to deeply analyze the unique immune profiles of the ovarian cancer tumor microenvironment and provide clues to the phenomenon that HRD tumors respond to PARPi rather than PD-1 immunotherapy. **b** Next, the authors pioneered a clinical trial of neoadjuvant PARPi for oral monotherapy of HRD advanced ovarian cancer (NANT, NCT04507841). **c** CCR8^+^ eTregs was then identified as a key responder using multi-modality profiling in clinical samples, emerging as a novel immunotarget for HRD ovarian cancer. **d** Anti-CCR8 mAb was successfully verified an effective immunotherapy regimen, which pave the way for the more effective therapies and improving patient outcomes combined with niraparib. **e** The lower panel of the diagram illustrates the cyclical loop that connects clinical practice with basic research and back to clinical application, aiming to address key biological questions and to pioneer innovative therapeutic strategies. This process concludes the main four steps: (1) discover new biological insights based on the current clinical therapy; (2) design new clinical trials according to the current unsolved research questions; (3) identify key responders using clinical samples and multi-modality profiling; (4) enlighten novel immunotargets to help develop new clinical therapy

In a recent paper published in *Cell*,^1^ Luo and colleagues performed a multi-omics analysis of a prospective phase II clinical trial and elucidated the effector regulatory T cells (eTregs) as novel immunotarget for ovarian cancer with homologous recombination deficiency (HRD), analyzing for the first time at the clinical level how poly (ADP-ribose) polymerase (PARP) inhibitors reshape the ovarian cancer microenvironment. The implications of targeting eTregs and combining PARP inhibitors could pave the way for more effective therapies clinically, which is also a typical example of practicing the concept of reverse transformation medicine (RTM) (Fig. 1).

Ovarian cancer (OC), the most malignant tumor in woman, leads to high mortality and low 5-year survival rates. Epithelial OC, which accounts for 90% OC cases, exists five major clinically and genetically distinct histotypes.^2^ The most common one is high-grade serous ovarian cancer (HGSOC), characterized with genomic instability with frequent *TP53* mutations and HRD. Multiple large randomized controlled trials (RCT) have confirmed that programmed cell death-(ligand)1 (PD-(L)1) inhibitory immunotherapy did not yield a survival benefit (NCT02718417, NCT02891824, NCT03038100, NCT02580058), but PARPi maintenance therapy benefits ovarian cancer patients, particularly those positive for HRD.^3^

Therefore, the current therapy for advanced ovarian cancer is primary cytoreductive surgery and standard first-line chemotherapy, and PARP inhibitor (such as Olaparib) is commonly used for first-line maintenance post-primary and recurrence chemotherapy, especially for patients with HRD-positive tumors. However, the precise impact of PARP inhibitors on treatment efficacy remains unclear. Understanding the unique characteristics of the HRD-driven tumor microenvironment and how PARP inhibitors reshape it are critical questions for further investigation. Additionally, the limited efficacy of PD(L)-1 inhibitors in ovarian cancer treatment highlights the need for innovative therapeutic approaches and novel targets for patients who are not candidates for surgery or develop resistance to platinum-based neoadjuvant chemotherapy (Fig. 1a).

To investigate the effects of PARPi, HRD and their interactions on the tumor microenvironment (TME), Luo and colleagues initiated a clinical trial of the neoadjuvant niraparib monotherapy (NANT, NCT04507841) in the treatment of advanced HRD ovarian cancer, patients who received neoadjuvant chemotherapy (NACT) were selected as controls. This cohort encompasses patients with both homologous recombination-proficient (HRP) and HRD status, aiming to record their different response to NANT and NACT. Multi-omic profiling was performed on tumors across sites and patient-matched blood samples, with key technical tools used including single-cell T cell receptor (scTCR-seq), bulk TCR seq, and multiplex immunohistochemistry (mIHC) (Fig. 1b).

First, clinical monitoring according to CA125 response criteria showed that NANT had significant therapeutic efficacy in HRD patients with the 73.6% response rate (RR) and a good safety profile. A specific cell-state hierarchy approach based on single-cell transcriptome sequencing revealed that numerous cell states exhibited concordant changes in response to both NANT and NACT, suggesting chemotherapy and niraparib treatment may act on the same cell population. Using scTCR-seq and co-analysis, eTregs were identified uniquely enriched in HRD-HGSOC and NANT, co-occurred with terminally exhausted CD8^+^ T cells (Texs). Interestingly, state transitions between eTregs and Texs in tumor-reactive T cells were orchestrated by HRD status and perturbed by NANT. The enrichment of eTregs in the HRD state significantly diminished after niraparib and platinum chemotherapy, aligning with a recent translational study showing marked reduction of eTregs in epithelial OC patients treated with PARPi maintenance.^4^ Using sc-RNA/TCR-seq data, clonal origins of limited expanded eTregs and highly expanded Texs were traced, which derived from CD4^+^ proliferative pool and effector CD8^+^ T cells, respectively. In terms of mechanism, the proportion of IFN-responsive tumor cells were significantly positively correlated with the proportion of eTregs in the tumor, suggesting that tumor cells may recruit or mediate eTregs activation through the high expression of type II MHC molecules and co-inhibitory molecules (Fig. 1c). Above findings underscore the key immunoregulatory role of eTregs in HRD-dependent and therapy-perturbed tumors, suggesting a potential improved therapeutic efficiency of targeting eTregs combined with niraparib especially in the HRD-context.

C-C chemokine receptor type 8 (CCR8) is recognized as a definitive phenotypic and therapeutic marker for eTregs. To test above hypothesis, Luo et al. first constructed a CCR8-humanized (h*CCR8*) mice and utilized humanized therapeutic monoclonal antibody (mAb) targeting CCR8 named ZL-1218, which is evaluated in a phase I clinical trial for advanced solid tumors currently (NCT05859464). Then an orthotopic mouse model relevant to human HGSOC was established by intrabursal injection of *Trp53*^−/−^ and *Brca1*^−/−^ ID8 cells, showing a higher proportion of eTregs and terminal Texs. Niraparib or CCR8 mAb used alone can attenuate tumor progression, but their combination yields a more pronounced inhibitory effect. Additionally, another Treg-depleting therapy using CD25 mAb demonstrated a similar therapeutic effect. The synergistic therapeutic combination of niraparib and either CCR8 or CD25 mAb has exhibited broader applicability and effectiveness in other orthotopic breast cancer mouse models with breast cancer susceptibility gene (BRCA) deficiency, which enlighten a novel immunotarget of eTregs for HRD ovarian cancers. Further evaluations of combination therapy with anti-CCR8 mAb and niraparib in advanced solid tumors, especially in HRD-positive or chemotherapy-resistant patients, could benefit more patients. Thus, this clinical-basic-clinical research loop was completed (Fig. 1d).

In summary, this study exemplifies the classic research paradigm of RTM.^5^ It begins with clinical observations of ovarian cancer patients’ responses to current therapy, discovering new biological insights. A well-curated patient cohort was then designed, utilizing multi-omics to dynamically characterize the treatment-perturbed microenvironment and patient outcomes. Key responsive cell populations and molecular targets were then identified based on the advanced technology and tools. Last, the preclinical efficacy of combination therapies targeting eTregs and niraparib was rigorously validated using humanized mouse models and humanized therapeutic mAb, which enlightens novel immunotargets. This process completes the cyclical loop from clinical practice to basic research and back to clinical application, which not only addresses key biological questions but also offers innovative therapeutic strategies for ovarian cancer (Fig. 1e).

Understanding the variability in human pharmacological responses is fundamental to RTM. Essential steps include dynamic detection, data sharing, and collaborative research. Monitoring the molecular and cellular dynamics in patients influenced by the treatment requires large-scale data integration and complex algorithms. These insights are fed back to basic researchers to uncover specific molecular mechanisms underlying tumor response or resistance, thereby allowing for the refinement of existing theoretical models and the generation of more precise predictions regarding drug efficacy. Ultimately, this knowledge empowers clinicians to devise tailored treatment approaches, enhancing patient outcomes across various conditions.

This integrative approach underscores the power of RTM in bridging the clinical observations and fundamental biological insights, thereby driving the development of personalized medicine. This is not only significant for treating ovarian cancers but also holds promise for other advanced solid tumors.
